# Benthic studies adjacent to Sakhalin Island, Russia 2015 III: benthic energy density spatial models in the nearshore gray whale feeding area

**DOI:** 10.1007/s10661-022-10018-7

**Published:** 2022-10-18

**Authors:** Arny L. Blanchard, Laurie Ainsworth, Glenn Gailey, Natalia L. Demchenko, Ilya A. Shcherbakov

**Affiliations:** 1 Blanchard Ecological, North Pole, AK 99705 USA; 2 Phistat Research and Consulting, North Vancouver, BC Canada; 3 Cetacean EcoSystem Research, Lacey, WA 98512 USA; 4 A.V. Zhirmunsky National Science Center of Marine Biology, Far East Branch of Russian Academy of Sciences, Vladivostok, Russia

**Keywords:** Benthic ecology, Marine ecology, Ecosystem variability, Macrobenthos, Sea of Okhotsk

## Abstract

Energy densities of six dominant benthic groups (Actinopterygii, Amphipoda, Bivalvia, Cumacea, Isopoda, and Polychaeta) and total prey energy were modeled for the nearshore western gray whale feeding area, Sakhalin Island, Russia, as part of a multi-disciplinary research program in the summer of 2015. Energy was modeled using generalized additive mixed models (GAMM) with accommodations for zero-inflation (logistic regression and hurdle models) and regression predictions combined with kriging to interpolate energy densities across the nearshore feeding area. Amphipoda energy density was the highest nearshore and in the south whereas Bivalvia energy density was the highest offshore and in the northern portion of the study area. Total energy was the highest in mid-range distances from shore and in the north. Amphipoda energy density was higher than minimum energy estimates defining gray whale feeding habitats (312–442 kJ/m^2^) in 13% of the nearshore feeding area whereas total prey energy density was higher than the minimum energy requirement in 49% of the habitat. Inverse distance-weighted interpolations of Amphipoda energy provided a broader scale representation of the data whereas kriging estimates were spatially limited but more representative of higher density in the southern portion of the study area. Both methods represented the general trend of higher Amphipoda energy density nearshore but with significant differences that highlight the value of using multiple methods to model patterns in highly complex environments.

## Introduction

Variations in water circulation, seafloor topography, and/or coastline geomorphology interacting with carbon delivery and benthic biological processes can create hotspots of energy accumulation in marine systems (Blanchard & Feder, 2014; Feder et al., 2007; Grebmeier et al., 2015). The spatial characteristics of benthic communities can be controlled through variations in the processes, volumes, and quality of carbon delivered to the benthos driven by water current characteristics, nutrient exchange, stability of water masses, and seasonal oceanographic patterns. Oceanographic characteristics (nutrient concentrations, stratification, and related variables) largely define the amount and type of carbon produced while seafloor and coastal landscape features such as submerged canyons, points, and straits and oceanographic features influence how and where carbon is deposited. Topographic features can, however, modulate oceanographic conditions leading to altered production patterns such as in polynyas (Ambrose & Renaud, 1995). As a result, large marine topographic features can be associated with long-term concentrations of organic carbon and accumulations of energy in benthic systems (Ambrose & Renaud, 1995; Buhl-Mortensen et al., 2012; Grebmeier et al., 2015). Temporally, macroscale climate patterns may indirectly influence benthic communities through control of broad-scale circulation patterns influenced by, among other things, baryotropic circulation, freshwater discharges, and winds (Blanchard, 2015; Blanchard et al., 2019; Kim, 2012). Temporally persistent benthic energy concentration hotspots result in reliable prey resources for benthic-feeding marine mammals and have great ecological importance (Blanchard & Feder, 2014; Blanchard et al., 2019; Brower et al., 2017; Grebmeier et al., 2015; Pisareva et al., 2015; Ray et al., 2006).

The drivers of complex ecological interactions are difficult to identify and measure. Inferences can be especially challenging in open marine systems where it is not possible to measure all components needed for modeling abiotic/biotic associations. Imbalances in designs, lack of repeated sampling events, mismatches between scales of sampling, drivers and interactions, and incomplete data collections lower power for detecting ecologically important patterns and can critically limit inferences (Blanchard & Feder, 2014). In the context of modeling and predicting benthic characteristics, digital elevation models (DEMs) can provide cost-effective alternatives for describing abiotic/biotic associations in the absence of measured environmental covariates (Huang et al., 2011; McArthur et al., 2010). DEMs can match landscape-scale patterns while also capturing small-scale complexity that affects benthic assemblages, and can successfully predict changes in biological communities when appropriate indices are used (Beatty et al., 2016; Choi et al., 2011; Knouft et al., 2011; Lee et al., 2013; Sbrocco & Barber, 2013). DEM variables often covary with and can thus serve as proxies for habitat-related complexity associated with ecological interactions, such as prominent coastline characteristics causing current variations that concentrate food for the benthos and influence sediment grain-size.

Seismic surveys to map subsurface geological characteristics have been periodically conducted near the western gray whale (*Eschrichtius robustus*) feeding ground adjacent to the northeastern coast of Sakhalin Island, Russia, since 1996 (Bröker et al., 2015; Johnson et al., 2007; Weller et al., 2006). Changes in gray whale behavior and distribution due to sound exposure were reported during earlier seismic surveys (Bröker et al., 2015; Gailey et al., 2007; Weller et al., 2006; Yazvenko et al., 2007). Data were not, however, available to evaluate the potential influence of prey resource availability on whale responses (Gailey et al., 2016; Muir et al., 2016). Likewise, how changes in behavior or distribution would affect energy intake by whales had not been investigated. Changes in energetic intake would be particularly important for reproductive females (Villegas-Amtmann et al., 2015, 2017). It is not clear whether recent declines in prey biomass adjacent to Sakhalin Island in the western gray whale feeding ground (Blanchard et al., 2019; IUCN, 2019) might result in altered whale distributions, behavior, response patterns, or energy intake, nor is it clear how prey biomass declines might change whale responses to acoustical stress if distributions are conditioned on nearshore prey resources.

In 2015, multiple seismic surveys were planned adjacent to the nearshore portion of the western gray whale feeding ground, prompting development of a comprehensive monitoring program with the purpose of assessing impacts on gray whale behavior, distribution, and energetics from sound exposure and vessel presence (Aerts et al., 2022; Gailey et al., 2022; Schwarz et al., 2022). Blanchard et al. (2022) determined that biomass concentrations of some prey groups in 2015 declined from early and mid-summer to late summer and fall. Total prey biomass concentrations, however, were largely homogenous (differences were statistically indistinguishable) within the nearshore feeding area. Communities varied with water depth and sediment type with amphipods numerically dominant in shallower water with finer sediments and bivalves dominant in deeper water with coarser sediments (Blanchard et al., 2022; Demchenko & Fadeev, 2011; Sobolevskii et al., 2000).

In this paper, we test the hypothesis that sampling period, water depth, and geographic/DEM variables were significant predictors of energy density for dominant benthic fauna within the western gray whale nearshore feeding area. Spatial regression was conducted to test the hypotheses with adjustments for zero-inflation using hurdle models. Regression kriging was then used to interpolate energy distributions. The roles of environmental and physical complexity of the study area in defining characteristics of the regressions and interpolations of benthic energy density are discussed. The spatial regression modeling of benthic energy distributions using DEM variables provides complementary insights to the univariate and multivariate analyses documenting community trends for key organisms (Blanchard et al., 2022).

## Materials and methods

### Study area

**Fig. 1:**
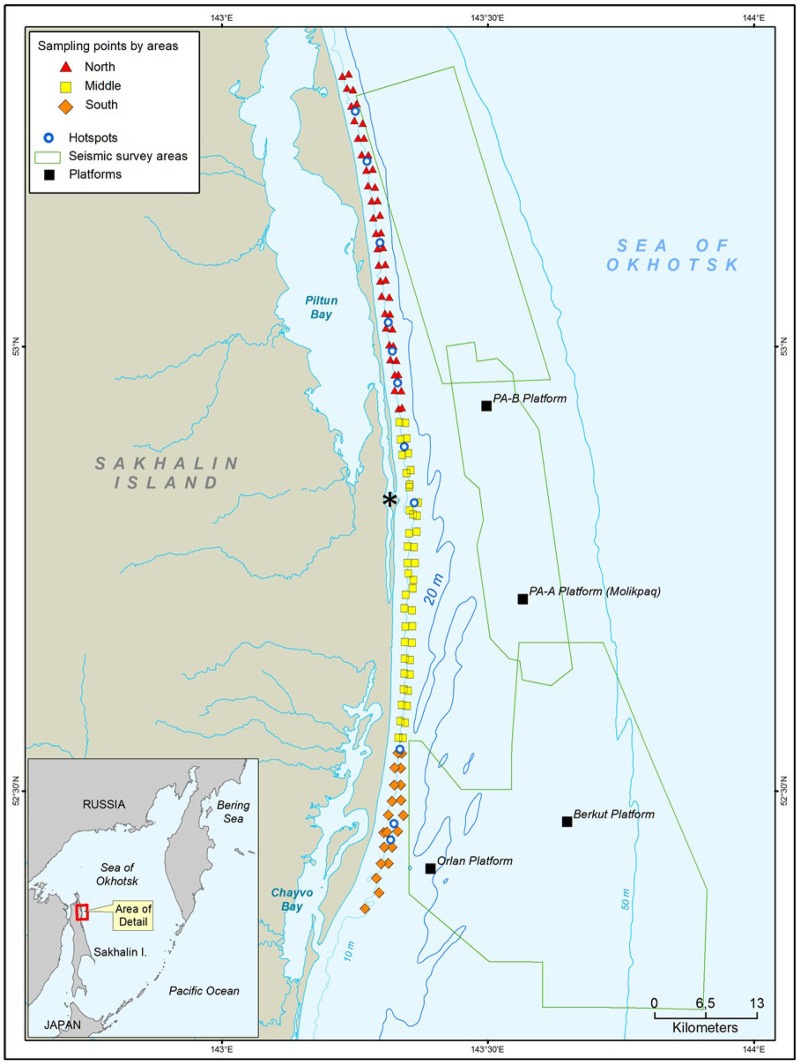
The 2015 benthic nearshore study area in the Sakhalin Island gray whale feeding area, Russia. The inset presents the geographic reference of the gray whale feeding area. See Aerts et al. (2022) for further details of the 2015 seismic mitigation and monitoring plan. The * marks the mouth of Piltun Bay

The western gray whale feeding ground is located off the northeastern coast of Sakhalin Island, Russia (Fig. 1). The study area comprises ~ 200 km^2^ of the nearshore feeding area as represented by a 95% contour of gray whale densities that encompasses ~ 600 km^2^ (Muir et al., 2015). Briefly, the northeastern Sakhalin coast is influenced in summer by freshwater from the Amur River via circulation around the northern tip of Sakhalin Island and outflows from Piltun Bay along the eastern Sakhalin shoreline. The nearshore feeding area is a turbulent, shallow water environment with strong environmental gradients and currents (Rutenko & Sosnin, 2014; SEIC, 2003). The macrobenthic community in the predominantely sandy sediments along the Sakhalin Island shoreline is influenced by strong ecosystem seasonality as well as by the physical dynamics of the nearshore area. Habitat characteristics include north to south environmental gradients associated with the southward circulation of Amur River outflows through the area in summer. In winter, the Eastern Sakhalin Current, the Sakhalin polynya, and sea ice cover influence circulation. The mouth of Piltun Bay is a point of change and increased complexity along the gradient in terms of bathymetry and ecosystem characteristics. The biomass-rich sediments in the study area are dominated by the amphipods *Eogammarus schmidti*, *Monoporeia affinis*, and *Anisogammarus pugettensis*, bivalves, the isopods *Saduria entomon* and *Synidotea cinerea*, and the sand lance *Ammodytes hexapterus* (Blanchard et al., 2022).

### Study design

Benthic biomass was sampled in the nearshore gray whale feeding area in summer, 2015 (Fig. 1). An intensive sampling program (Blanchard et al., 2022) was carried out encompassing three different geographic strata (North, Middle, and South Zones) during three sampling periods aimed at characterizing spatial and seasonal variation in benthic biomass. In addition to the detailed sampling, targeted benthic sampling was conducted in areas of observed whale feeding activity. Benthic samples collected from nearshore locations as part of a long-term survey were included when within or adjacent to the sampling grid (Blanchard et al., 2019; Demchenko, 2010; Fadeev, 2011, 2013a, 2013b). Three replicate grabs were collected at each station of the intensive (*n* = 223 stations) and long-term (*n* = 5) surveys while 12 grabs were collected in targeted sampling locations (*n* = 12). The distance from shore of sampling locations ranged from 0.86 to 3.56 km and water depth ranged from 6.7 to 16.1 m. Further details on benthic sampling methodology are discussed in Blanchard et al. (2022).

The biological data matrix included wet tissue biomass concentrations (g/m^2^) and conversions from biomass concentrations to energy density (kJ/m^2^) of the six benthic groups that dominated benthic biomass (Actinoptygerii, Amphipoda, Bivalvia, Cumacea, Isopoda, and Polychaeta) and total energy (the sum of energy density of the 6 groups; kJ/m^2^) as determined by bomb calorimetry (Maresh et al., 2022). Biomass was converted to energy density using multipliers of 5.2 for Actinoptygerii; 5.2 for Amphipoda; 4.0 for Isopoda; 2.3 for Bivalvia; 4.8 for Cumacea; and 4.4 for Polychaeta. Classification factors from the study design included zone (North, Middle, and South) (Fig. 3) and period (Period 1: 19 June to 15 July; Period 2: 16 July to 31 August; and Period 3: 1 September to 24 October). Dates were slightly modified from Blanchard et al. (2022) to accommodate the hotspot and long-term survey sampling that were placed into the closest detailed grid sampling period. Zone was confounded with the continuous geographic variable northing (UTM, m north representing distance alongshore from south to north) and was not included in regression models but was used as a classification factor for energy density plots. Tide-corrected water depths (m) and distance from shore (km) were determined from a GIS-based digital elevation model (DEM) (Choi et al., 2011; Lee et al., 2013). Other DEM variables (terrain, slope aspect, and roughness) were collinear with northing, distance to shore, or depth and were excluded.

### Statistical approach

The physical and environmental complexity of the study area made use of standard geostatistical interpolation methods such as universal kriging inappropriate as regression relationships were of a form requiring nonparametric smoothers. Instead, a multi-step analysis was conducted that included nonparametric regression modeling to fit complex predictors. As applied here, regression is statistical modeling with continuous and categorical predictors and continuous response variables with the characteristics of the regression model based on assumption testing through residual analysis. Nonlinear data relationships that do not fit a specific curve (multiple humps, very sharp changes, etc.) are data characteristics that can be addressed via nonparametric smoothing. Thus, the data features and residual analyses guided us to the specific form of regression applied (Zuur et al., 2009). We then conducted regression, kriging interpolation of regression residuals, model validations, and compilation of regression and kriging predictions to complete interpolated maps of benthic resources. Each of the steps is discussed separately.

#### Generalized additive mixed modeling

Generalized additive models (GAM) are generalized linear models using nonparametric smooth functions for predictors (Guisan et al., 2002; Yee & Mitchell, 1991). As a form of nonparametric regression, GAMs allow modeling of functional regression relationships (e.g., data-driven, smoothed relationships) among covariates, a particularly useful method for modeling biological processes (Ciannelli et al., 2008; Robertson et al., 2015). Generalized additive mixed models (GAMM) include random effects in a mixed model setting and were used here (Zuur et al., 2009). Regression models (GAMMs) were adapted as needed to accommodate the large proportion of zeros in the benthic biomass data. If 10% or less of biomass values were recorded as zero, a GAMM was used to determine regression models (unconditional regression) for biomass. Hurdle models were used to accommodate zero-inflation when more than 10% but less than 50% of the biomass values were zero for a group (Barry & Welsh, 2002; Cunningham & Lindenmayer, 2005; Lyashevska et al., 2016; Welsh et al., 1996). The hurdle model approach included two regression components: (i) a logistic GAMM of occupancy (regression of presence/absence data with a binomial error) and (ii) a GAMM of the characteristic when present (conditional regression). Final unconditional predictions for the hurdle models were calculated as the product of the predicted probability of occurrence and the predicted conditional biomass at each prediction location. Two-step regression (hurdle) models have been applied elsewhere in marine ecology for zero-inflated data (Ciannelli et al., 2008; Murase et al., 2009). If more than 50% of biomass values were zero, a logistic GAMM was fit to predict the probability of occurrence because there was not enough information in the positive biomass values to model.

Statistical models in this study included combinations of period, depth, distance to shore, and northing as predictor variables. Period was included in all models as a fixed factor covariate whereas splines (piece-wise polynomials as smoothed predictor values) were applied to water depth, distance to shore, and northing in GAMMs (Zuur et al., 2009). Station was included as a random effect. Two and three-way interactions were evaluated in the GAMMs via tensor products (Berhane et al., 2008; Wood, 2006). Tensors are products of splines of individual predictors permitting the evaluation of higher-order interactions not possible with standard regression techniques while also maintaining orthogonality and optimal convergence rates (e.g., prevents collinearity problems and convergence issues) as well as additivity. GAMMs using tensor products were applied here to flexibly fit the curvilinear relationships resulting from the shoreline topography and bathymetric complexity across the study area. For computational stability, northing was divided by 1000. Some replicate locations had indistinguishable coordinates and coordinate pairs were offset slightly (jittered). Water depth and distance from shore resulted in high variance inflation factor (*VIF*) values in preliminary regression diagnostics (*VIF* > 7). Thus, these latter two variables were not included in the same models but were tested separately.

Akaike’s Information Criteria (*AIC*) was used to select best-fitting models. A convenient means to rank regression models is the difference between a particular model’s *AIC* value and the minimum *AIC* for all models of an associated analysis (Δ_*AIC*_ = *AIC* − *AIC*_min_). As a rule of thumb, a Δ_*AIC*_ of 2 or less suggests the information captured by a model is equivalent to that of the lowest *AIC* model (Burnham & Anderson, 2002). Δ_*AIC*_ values between 3 and 7 indicate that a model captures less information than the model with minimum *AIC* and a model within this bracket may be selected if it provides substantially greater simplicity. An Δ_*AIC*_ of more than 10 indicates that the model does not fit the data well, compared to the model with the lowest *AIC*. Selection between models with similar *AIC* values (Δ_*AIC*_ ≤ 2) was based on the principal of parsimony; the simpler model was chosen.

Initial model fitting revealed uncharacteristically large predictions for locations outside the range of observed distances from shore. Thus, distance from shore and depth were truncated to balance fitting within the range of observed data and extrapolating to locations on the margins of the study area. Truncation distances were determined by a sensitivity analysis; all distance from shore values below 0.75 km were set to 0.75 km and all values above 4 km were set to 4 km for regression and kriging predictions. Similarly, water depth was truncated at 6 m and 18 m. Energy values were *ln*(*X* + 0.5)-transformed prior to analyses.

For marine benthic studies, effect sizes (*f*) of small (*f*_small_ = 0.2, percent variance accounted for or *PV* ≈ 4%), medium (*f*_medium_ = 0.5, *PV* ≈ 23%), and large (*f*_large_ = 0.8, *PV* ≈ 40%) were proposed for evaluation of chemical and physical disturbance to benthic communities (Blanchard et al., 2002; Cohen, 1988; Murphy et al., 2014). Here, the proportion of deviance explained, the analog for GAMM models of the coefficient of determination *R*^2^, was compared to the *PV* criteria above to approximate an effect size for the models. For the present situation, effects were approximated as a small effect exceeding *PV* = 0.04, a medium effect exceeding *PV* = 0.23, and a large effect exceeding *PV* = 0.40. The nonparametric smoothing used in GAMMs does not produce standard metrics of effect sizes (regression coefficients, standard errors, and associated effect size confidence intervals) and the approach here provides a reasonable approximation for testing effect sizes. The proposed effect size criteria have been applied to the interpretation of correlations (Blanchard et al., 2003, 2019) and were also applied to ANCOVA models of raw biomass for the Sakhalin Island study area using minimum-effects hypotheses (Murphy et al., 2014) in Blanchard et al. (2022). Power analyses are presented in Blanchard et al. (2022).

#### Kriging and final interpolation maps

A regression kriging approach (Webster & Oliver, 2007) was applied for geostatistical interpolation (kriging) of energy densities to accommodate the complex data characteristics. The multi-step kriging process included (1) fitting of unconditional and logistic GAMMs as appropriate; (2) determination of residuals from each GAMM; (3) kriging of residuals; and (4) the summation of regression and kriging predictions for each prediction location for a given model. The addition of kriging predictions to regression predictions incorporated local effects due to spatial correlations. Spatial maps were directly available for unconditional and logistic regressions as summed predictions. An additional step was required for hurdle models where kriging predictions from the logistic and conditional biomass regressions were multiplied to get the unconditional kriging predictions, as was also done for regression predictions to determine unconditional predictions. The unconditional regression predictions were then added to unconditional kriging predictions to interpolate an unconditional energy map. Interpolations are presented for Amphipoda, Bivalvia, Isopoda, and total energy.

The shape of the study area, a narrow shelf running north to south, meant that spatial correlations were generally much stronger in the east/west direction associated with the sharp depth gradient moving offshore, a violation of the assumption of isotropy (equal correlations in all directions). Geographic coordinates were transformed to correct for the anisotropy by converting distance from shore to decimeters and northing to kilometers for use as spatial coordinates. Under the revised coordinate system, movement in the north/south direction corresponded to moving linearly up and down the coast at a similar depth. A prediction grid with 20-m spacing was used for interpolation. Spatial correlations of regression residuals were fit with an exponential model except for logistic GAMM residuals for Cumacea and Isopoda, which were fit with spherical models. Final regression kriging predictions are averaged at a scale of 1 km^2^ to match the whale density scale used in Gailey et al. (2022).

#### Regression model validation

Model choices were made based on residual analyses. The excess numbers of zero values for some biomass variables resulted in severe violations of assumptions. Where there were enough data to effectively model positive biomass values, a hurdle model was used. Otherwise, where there were too many zeros, a logistic regression was applied. The decision to use GAMMs for regression was based on the structures of the predictor variables that demonstrated shapes that could not be modeled using standard linear or nonlinear regression but required nonparametric smoothers. Model fits for all regressions were verified by residual analysis to validate homogeneity of variances and no patterns in residuals. Independence was not assumed, and kriging was applied to account for spatial dependencies.

GAMM-fitted values were compared to competing models with correlation analyses. Models used for comparison and validation against regression models were inverse distance weighting (IDW) for all models and unconditional regressions for hurdle models. IDW is commonly used to map data and provides a descriptive presentation based on the maximum distance and number of neighbors allowed for estimation. An advantage is that hotspots are readily visible but IDW can substantially increase prediction error (~ 20%) relative to kriging (Yasrebi et al., 2009). Regression models, on the other hand, can over-smooth areas with high complexity or give unreasonable values at gradient edges. Model efficiencies were assessed by comparing fitted values among IDW, hurdle, and unconditional GAMMs. Where hurdle models were used, fitted values from unconditional GAMMs were determined using period and a smoothed tensor product for northing and distance to shore for comparison against conditional biomass models. IDW-fitted values for observed locations were determined by cross-validation and cross-validation prediction errors were determined for GAMMs.

#### Kriging and IDW comparisons

Regression kriging and IDW interpolations were compared for Amphipoda energy to understand their comparative strengths. Whereas regression kriging is limited by sampling constraints (mismatches between predictor combinations over space and time causing numerical instability and very large prediction errors), IDW estimates are not. Kriging and IDW predictions were mapped for their appropriate domains for each sampling period and fits compared to the observed data to clarify the strengths of each approach.

#### Software

All regression and spatial models were fit and assessed using the statistical software R (R Core Team, 2019). The *gamm* function in the *mgcv* package (Wood, 2011) was used to conduct GAMM regression and the *aictab* function in the *AICcmodavg* package (Mazerolle, 2017) was used to assess the fit of regression models. Spatial modeling was conducted using the *geoR* (Ribeiro et al. 2020) and *gstat* packages (Gräler et al., 2016; Pebesma, 2004). ArcGIS 10.3 (Esri, 2011) was used to plot regression kriging components as IDW maps of spatial predictions. GAMM model cross-validation was conducted using *CVgam* in the *gamclass* package (Maindonald, 2018) and the function *gstat.cv* of the *gstat* package was used to cross validate IDW predictions.

## Results

### Regression

#### Unconditional regression

**Fig. 2:**
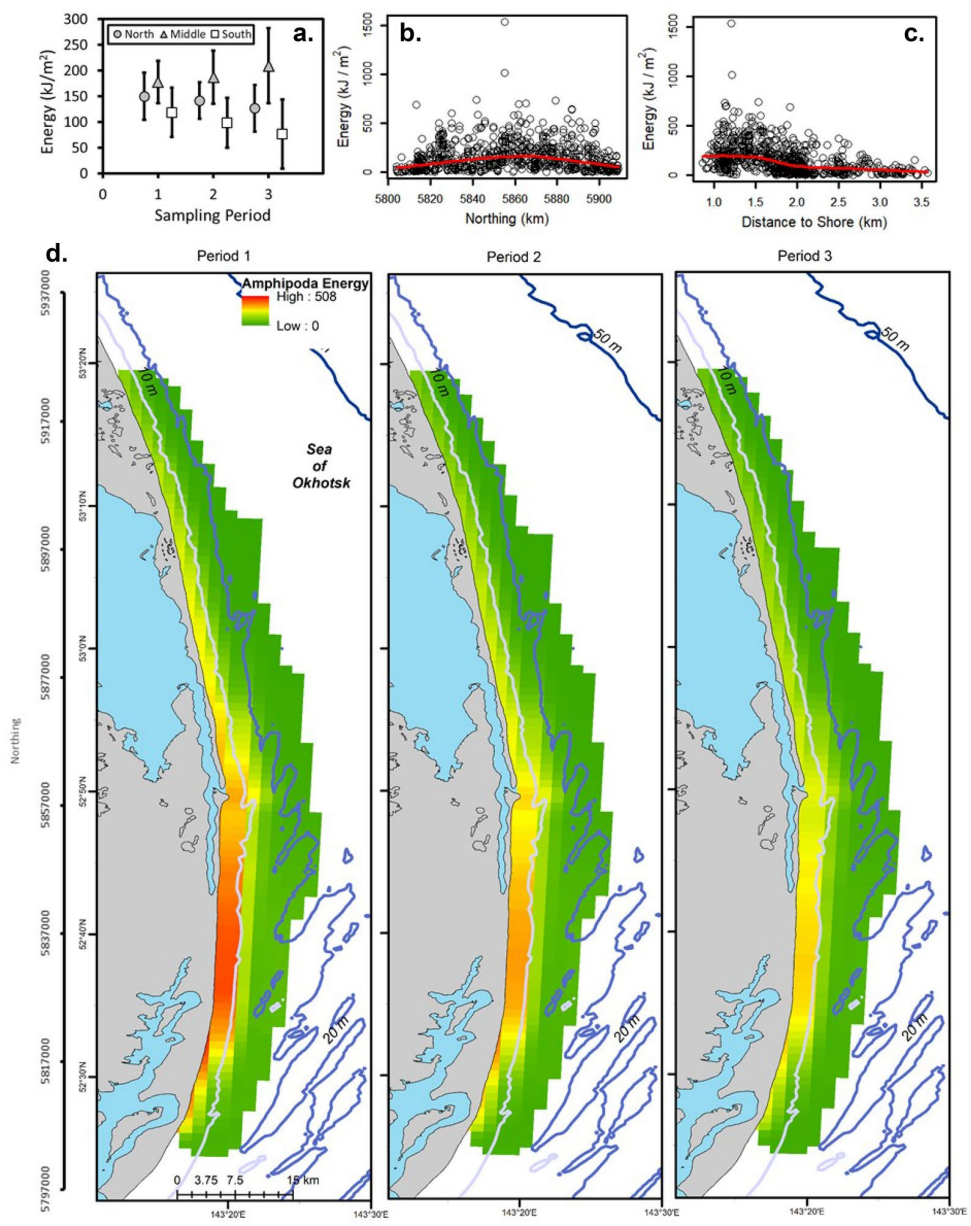
Amphipoda energy (kJ/m^2^) for the nearshore feeding area, Sakhalin Island Russia, 2015. Observed energy density is presented by **a** sampling period and zone, **b** densities by northing with a smoothed regression, and **c** densities by distance to shore with a smoothed regression. **d** Presents kriging predicted energy density by sampling period. Northing (m north) and latitude (degrees north) are presented on the vertical scale

**Fig. 3:**
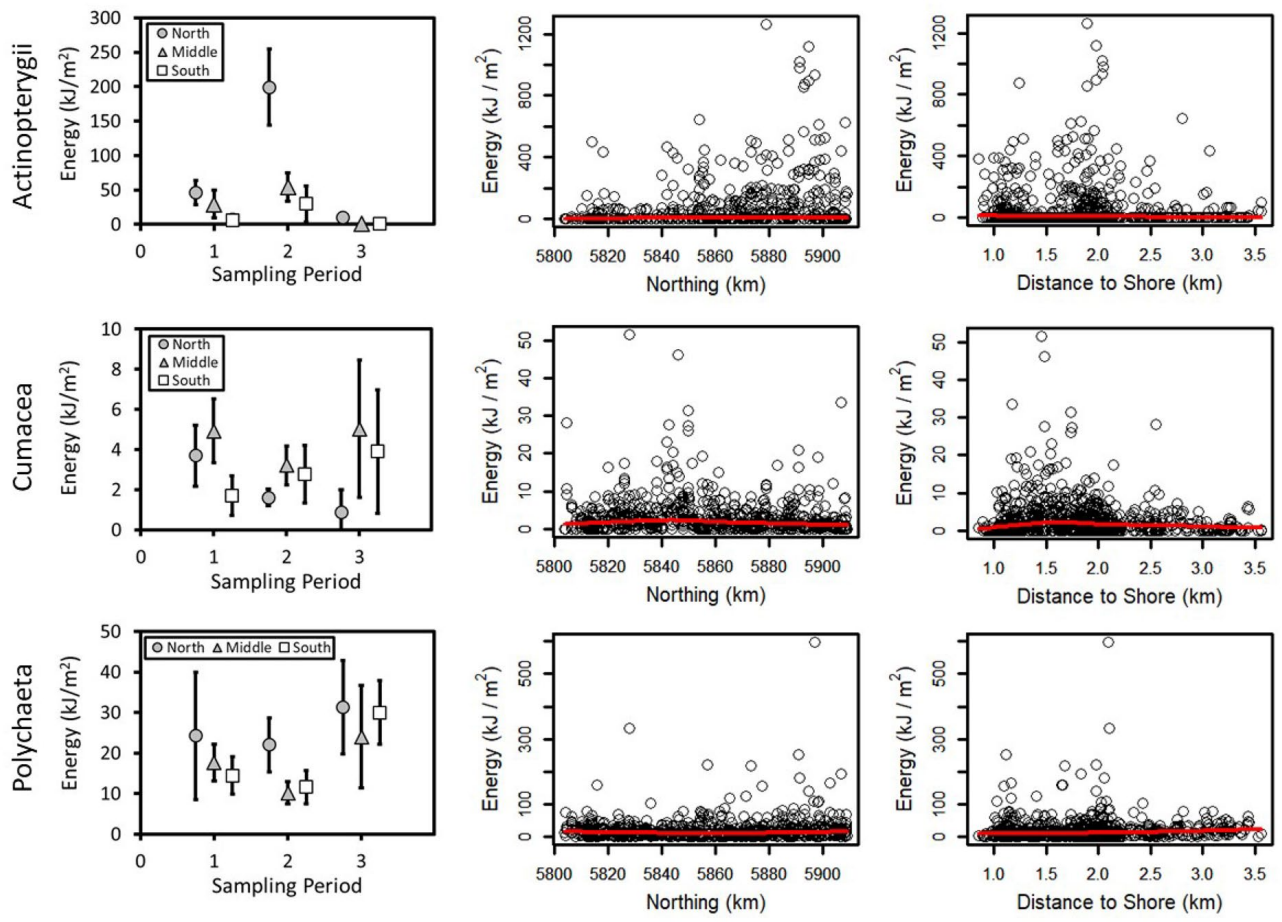
Average energy density (kJ/m^2^) by period and zone and scatterplots of northing (km) and distance from shore (km) for Actinopterygii, Cumacea, and Polychaeta for the nearshore feeding area, Sakhalin Island, Russia, 2015. Smoothed regressions are presented on scatterplots

**Fig. 4:**
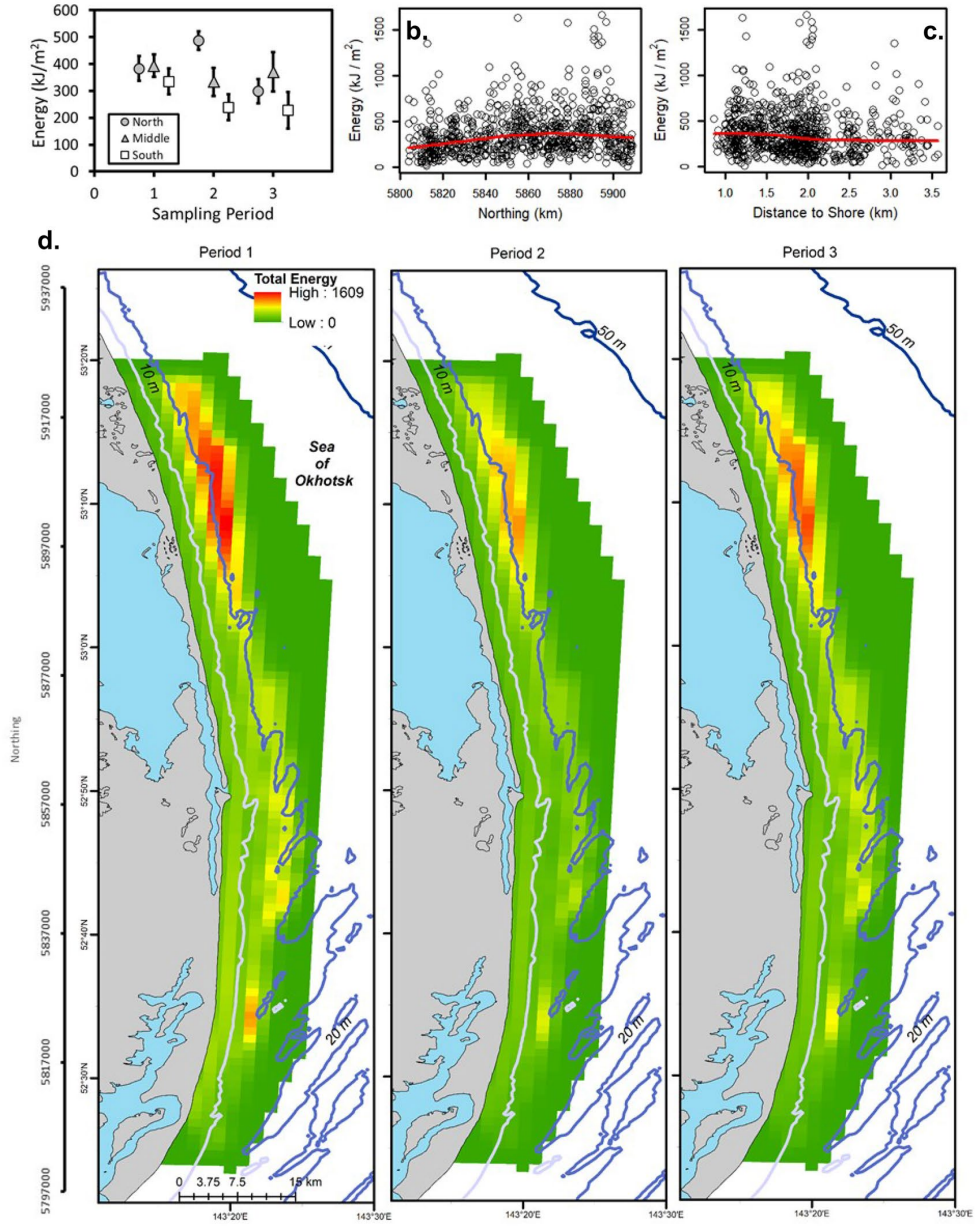
Total energy (kJ/m^2^) for the nearshore feeding area, Sakhalin Island, Russia, 2015. Observed energy density is presented by **a** sampling period and zone, **b** northing with a smoothed regression, and **c** distance to shore with a smoothed regression. **d** Presents kriging predicted energy density by sampling period. Northing (m north) and latitude (degrees north) are presented on the vertical scale

The unconditional GAMM (with all data) for Amphipoda (deviance explained = 0.53), Polychaeta (deviance explained = 0.16), and total energy (deviance explained = 0.15) included period and the northing by distance interaction (Tables 1 and 2). Amphipoda predicted energy density was the highest in Period 2 and lowest in Period 3 as indicated by multiple comparisons (Fig. 2; Table 2). Amphipoda energy density was also high at ~ 5,860 km northing and in shallower water depths. Polychaeta energy density was higher in Period 3 than Period 1 (Fig. 3; Table 2). Observed Polychaeta energy density increased slightly with higher northing values and was nearly constant with respect to distance from shore with a few points forming a peak at ~ 2 km. Total prey energy density increased with greater northing values and was slightly higher from 1 to 2 km from shore (Fig. 4; Table 2). Multiple comparisons demonstrated that total energy in Period 1 was significantly higher than Period 3. The regression for Amphipoda indicated a large effect (deviance explained ≥ 0.4) and small effect sizes were noted for Polychaeta and total energy density (0.04 ≤ deviance explained < 0.23).

**Table 1:** Biomass concentrations and energy density for dominant prey groups and the total for all six groups for stations used in regression from the nearshore study area, Sakhalin Island, Russia. Minimum and maximum values are calculated using replicate grabs. % total is percent average prey biomass of total prey biomass

Biomass (g m^−2^)	Mean	Median	Minimum	Maximum	% Total	Zeros (%)
Actinopterygii	10.6	0.0	0.0	243.0	10.8	67.7
Amphipoda	29.7	22.1	0.0	295.6	30.4	0.5
Bivalvia	46.1	19.1	0.0	557.5	47.2	17.5
Cumacea	0.6	0.3	0.0	10.8	0.6	26.2
Isopoda	6.4	2.9	0.0	117.2	6.5	10.7
Polychaeta	4.4	2.3	0.0	136.0	4.5	5.7
Total Biomass	97.7	80.9	1.7	572.5	n/a	0.0

Energy (kJ m^−2^)	Mean	Median	Minimum	Maximum	% Total	Caloric conversion
Actinoptygerii	55.1	0.0	0.0	1263.6	15.2	5.2
Amphipoda	154.2	114.9	0.0	1537.0	42.4	5.2
Bivalvia	106.1	44.0	0.0	1282.3	29.2	2.3
Cumacea	2.9	1.4	0.0	51.6	0.8	4.8
Isopoda	25.6	11.4	0.0	468.8	7.0	4
Polychaeta	19.2	9.9	0.0	598.4	5.3	4.4
Total -Energy	363.7	307.5	6.6	1662.8	n/a	n/a

**Table 2:** Best-fit regression models for benthic prey from Sakhalin Island, Russia, 2015. *F* statistics and *p* values are presented. *F* statistics are the first value in each cell followed by the *p* values. Smoothed terms were estimated for all variables and interactions except for the simple effect of period. *Dev*, deviance explained; *Gs*, GAMM-scale; *Xv*, cross-validation error; *D%*, percent difference between Xv and Gs; *N*, northing; Dist, distance to shore; and Per, period. *Logist*, logistic regression; *Uc Reg*, unconditional regression; and *Con Reg*, conditional regression (positive biomass values only). Act, Actinopterygii; *Amp*, Amphipoda; *Biv*, Bivalvia; *Cum*, Cumacea; *Iso*, Isopoda; and *Pol*, Polychaeta

Group	Model	Dev	Gs	Xv	D%	Period	MC	Northing	Distance	Depth	N*Dist	N*Per
Act	Logist	0.23	0.17	0.18	3.0	35.4, < 0.0001	P3 < P1 < P2	–	–	–	12.0, < 0.0001	–
Amp	Uc Reg	0.56	0.49	0.51	3.5	14.1, < 0.0001	P3 < P1 < P2	–	–	–	38.9, < 0.0001	–
Biv	a) Logist	0.16	0.12	0.12	1.2	3.4, 0.0327	ND	–	–	26.8, < 0.0001	–	–
	b) Con Reg	0.30	1.56	1.66	6.5	5.2, 0.0052	ND	9.1, < 0.0001	–	158.0, < 0.0001	–	–
Cum	a) Logist	0.10	0.18	0.18	1.8	10.9, < 0.0001	P3 < P1, P2	4.6, 0.0003	–	–	–	–
	b) Con Reg	0.27	0.12	0.12	4.1	8.0, < 0.0001	P2 < P1	8.9, < 0.0001	20.2, < 0.0001	–	–	–
Iso	a) Logist	0.17	0.08	0.08	1.9	3.9, 0.0204	ND	23.2, < 0.0001	–	–	–	–
	b) Con Reg	0.48	0.48	0.53	9.3	P1*	–	–	–	–	–	15.6, < 0.0001
						P2	–	–	–	–	–	8.0, < 0.0001
						P3	–	–	–	–	–	9.0, < 0.0001
Pol	Uc Reg	0.16	0.62	0.65	4.7	16.4, < 0.0001	P1, P2 < P3	–	–	–	9.2, < 0.0001	–
Total	Uc Reg	0.15	0.41	0.43	3.6	7.7, < 0.0001	P3 < P1	–	–	–	8.1, < 0.0001	–

*Interactions of period with the tensor product of northing and distance from shore are presented as *F* statistics for each period

#### Logistic regression

A logistic GAMM (regression of presence/absence data) was conducted for Actinopterygii, the group for which the proportion of zero biomass values exceeded 60% (Table 1). The best-fitting model for Actinopterygii included period and the northing by distance interaction via a tensor product (deviance explained = 0.23; Table 2). Observed Actinopterygii energy density declined from north to south across all periods and multiple comparisons indicated that Period 2 was significantly higher than Period 1 and Periods 1 and 2 were significantly higher than Period 3 (Fig. 3; Table 2). Actinopterygii energy density increased with northing and water depth and peaked ~ 2 km from shore. The logistic regression for Actinopterygii energy density represented a small effect.

#### Hurdle models

**Fig. 5:**
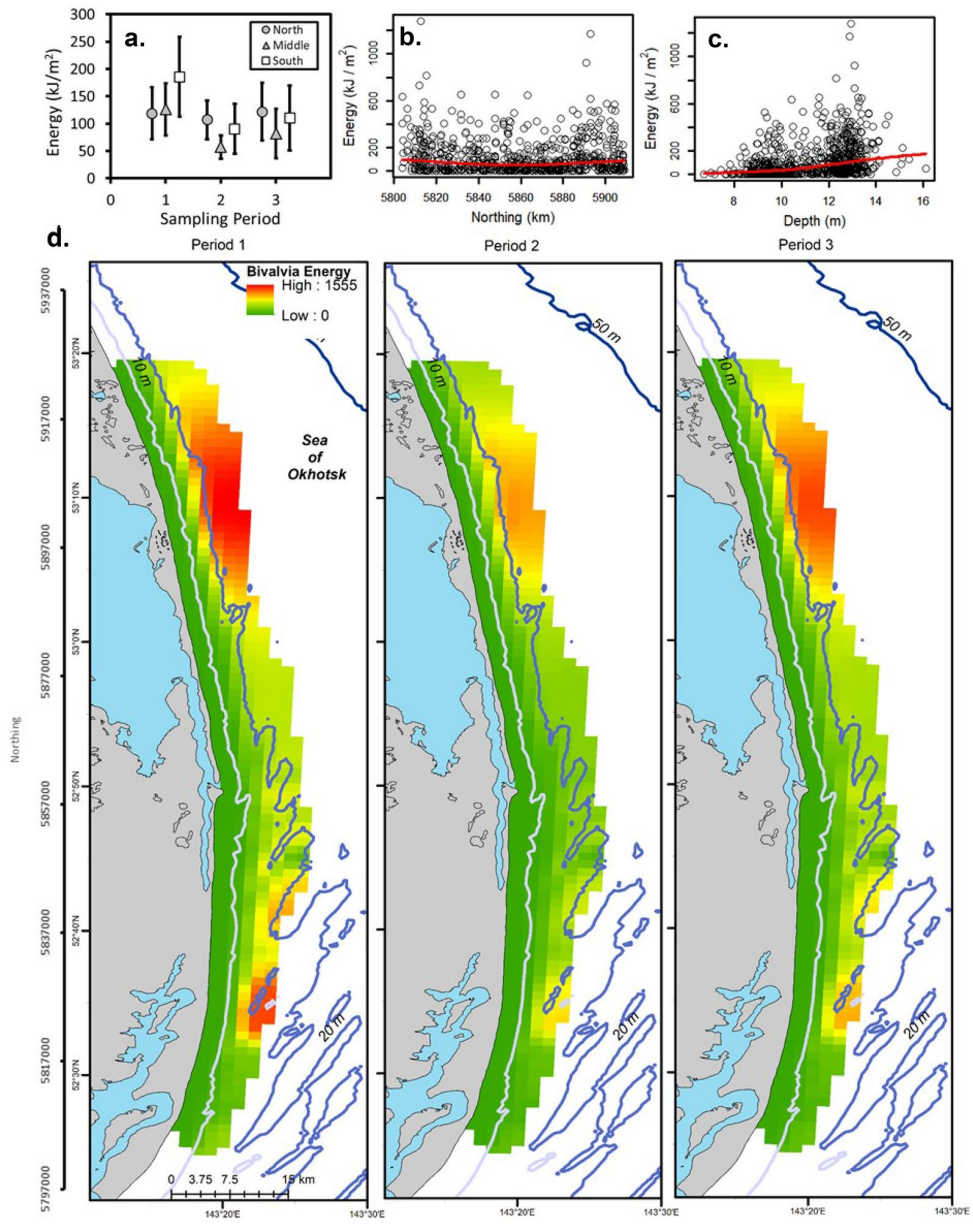
Bivalvia energy (kJ/m^2^) for the nearshore feeding area, Sakhalin Island Russia, 2015. Observed energy density is presented by **a** sampling period and zone, **b** northing with a smoothed regression, and **c** depth with a smoothed regression. **d** Presents kriging predicted energy density by sampling period. Northing (m north) and latitude (degrees north) are presented on the vertical scale

**Fig. 6:**
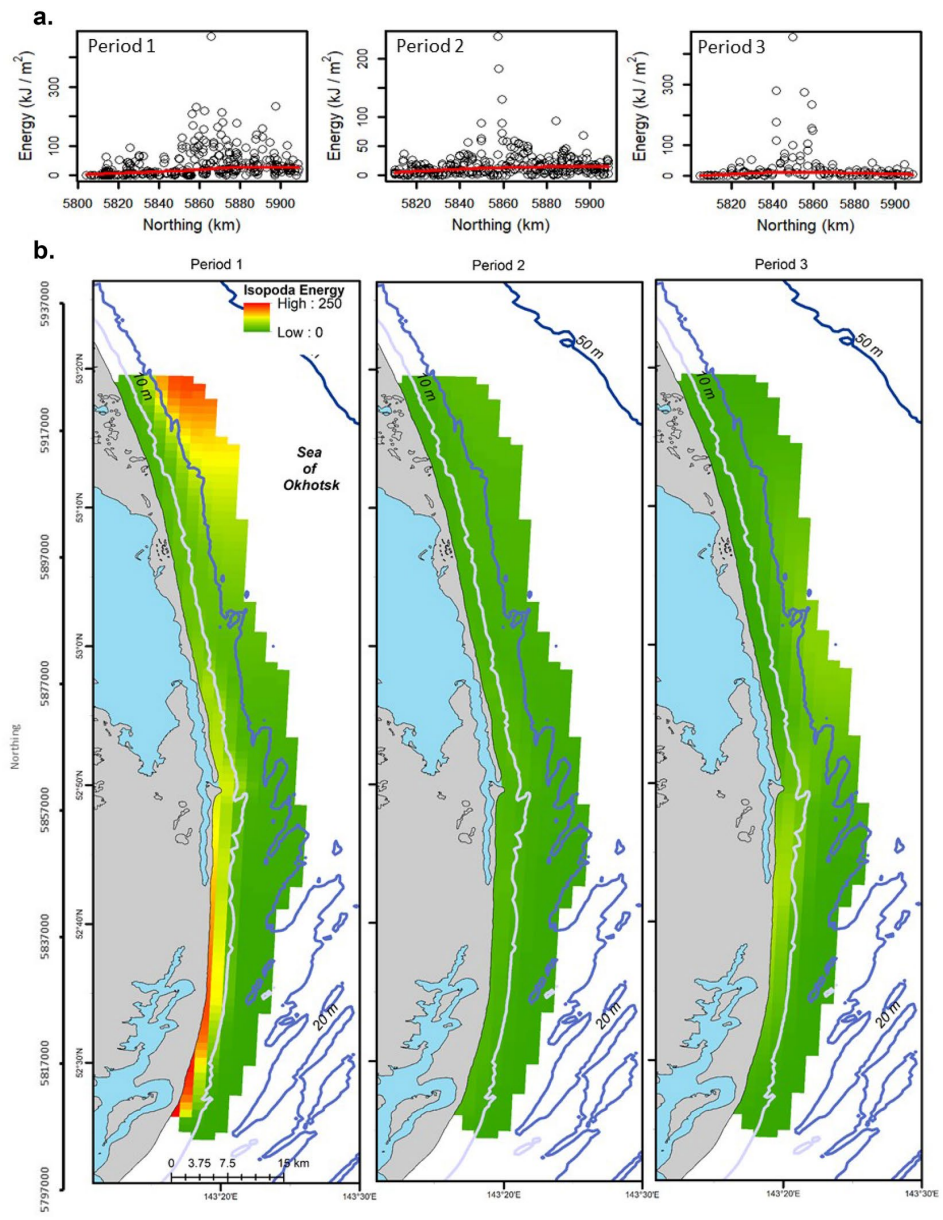
Isopoda energy (kJ/m^2^) for the nearshore feeding area, Sakhalin Island Russia, 2015. Observed energy density is presented by **a** northing and sampling period with smoothed regressions. **b** Presents kriging predicted energy density by sampling period. Northing (m north) and latitude (degrees north) are presented on the vertical scale

Hurdle GAMMs were fit for Bivalvia, Cumacea, and Isopoda. The best-fitting logistic and conditional GAMM regressions for Bivalvia energy included period and water depth (deviance explained = 0.16 and 0.30; Fig. 5; Table 2). Observed Bivalvia energy density was the highest in Period 1 in the South Zone and lowest in the Middle Zone in Period 2 with no differences noted among periods in multiple comparisons. Bivalvia energy increased with water depth. The best-fitting logistic model for Cumacea energy included period and northing with the addition of distance for the conditional GAMM (deviance explained = 0.10 and 0.27; Table 2). Observed Cumacea energy density demonstrated low values in the south in Period 1 and in the north in Periods 2 and 3 while multiple comparisons demonstrated that Cumacea energy density was higher in Period 1 and 2 than in Period 3 (Fig. 3). Cumacea energy was nearly constant across all values of northing with a slight increase around 5,840 km north and was higher closer to shore. The best predictors of Isopoda occupancy were period and northing while the interaction of northing and period was the best predictor of conditional Isopoda energy (deviance explained = 0.17 and 0.48; Table 2). Isopoda energy density increased with greater northing values in Period 1 and peaked at ~ 5,480–5,860 km north in Periods 2 and 3 (Fig. 6). No differences were detected in multiple comparisons for period in the logistic GAMM for Isopoda though each element of the 3-way tensor product interaction was significant for the conditional GAMM (Table 2). The effect sizes for the hurdle model regressions were small for logistic regressions with Bivalvia and Cumacea conditional GAMMs having medium-sized effects (0.23 ≤ deviance explained < 0.4) and Isopoda conditional GAMM having a large-sized effect.

### Interpolation

Amphipoda energy densities were the highest in period 1, close to shore in shallow water, and south of the mouth of Piltun Bay (Fig. 2). Interpolations compare well with observed patterns as Amphipoda energy density declined from Period 1 to Period 3 and with greater distance from shore though the peak in energy in the middle of the study area in Period 3 from the observed data was not captured in the interpolations. Scatterplots of observed Amphipoda energy density suggested that value peak in the middle of the study area and predicted energy density was the highest just south of the mouth of Piltun Bay.

Total energy density was the highest in Period 1 and to the north and declined over the study period (Fig. 4). Predicted total energy increased with northing, declined with greater distance to shore, and was the highest in the northern portion of the study area where Actinopterygii energy was high.

Bivalvia predicted energy concentrations were the highest in deeper water farther from shore and in the southern and northern portions of the study area (Fig. 5). Bivalvia energy predictions (maximum value of 1,555 kJ/m^2^) were the highest along the eastern (deeper) edge of the prediction grid where they exceeded the observed maximum (1,282 kJ/m^2^; Table 1) representing an overestimation of Bivalvia energy along the eastern boundary.

Isopoda energy density was predicted to be highest in Period 1 nearshore in the south and farther from shore in deep water in the northern portion of the study area (Fig. 6). Predicted Isopoda energy density declined over the study period with low density across the whole study area in Periods 2 and 3.

### Regression model validation

Fitted energy densities were similar to observed averages for most models with some exceptions (Table 3). Actinopterygii probability of occurrence and fitted energy density values increased where observed data were high in the north zone in Period 1 and particularly in Period 2. Amphipoda energy density fitted values closely followed the peak in energy in the middle of the study area but declined over time whereas observed energy increased in the middle area across periods. The unconditional and hurdle (without correction for zero-inflation) model fitted values for Bivalvia energy were all lower than the observed data suggesting a downward model bias. Cumacea, Isopoda, Polychaeta, and total energy density fitted values were generally similar to but slightly lower than the observed data. Overall, IDW energy density fitted values were close to the observed data and usually the closest to the observed means of all the methods. IDW-fitted values had the highest correlations with observed data (Spearman’s $\rho$ ranged from 0.91 to 0.98) for all groups indicating that IDW maps were more faithful to observed patterns. GAMM-fitted values for Amphipoda were highly correlated with the observed data ($\rho$  = 0.81) and had the highest correlation for GAMMs among all groups (0.47 ≤ $\rho$≤ 0.72 for other groups). Hurdle model fitted value correlations with the observed data ranged from $\rho$ = 0.61 to $\rho$ = 0.73 for Bivalvia, Cumacea, and Isopoda. The unconditional GAMM for Bivalvia had a slightly higher correlation with the observed data than fitted values from the hurdle model, despite the accommodation of zero values in the latter method. GAMM predictions of Polychaeta and total energy had lower correlations, with values of $\rho$ = 0.47 and 0.48. The latter two groups comprise multiple trophic guilds and life histories and lower fits can be expected given the narrow ecological conditions in the study area and wide environmental tolerances, particularly for polychaetes. Cross-validation errors for each GAMM model were less than 10% of model error and for the most part less than 5% (Table 2).

**Table 3:** Means (kJ/m^2^) of observed data and fitted values for GAMMs and IDW by period (Per) and sampling strata (zone: Zn) for Sakhalin Island, Russia, 2015. *Data*, observed data; *Hur*, hurdle model; *GM*, unconditional GAMM; *IDW*, inverse distance weighting; *Act*, Actinopterygii; *Amp*, Amphipoda; *Biv*, Bivalvia; Cum, Cumacea; *Iso*, Isopoda; *Poly*, Polychaeta; and *Total*, total energy. Correlations are between fitted values and observed data

Group	Per	Zn	Data	Hur	GM	IDW	Group	Per	Zn	Data	Hur	GM	IDW
Act.*	One	S	5.2	0.1	0.5	5.4	Cum	Two	N	1.6	1.5	1.4	1.5
		M	28.6	0.2	4.3	32.6		Three	S	3.9	1.6	1.9	4.1
		N	45.3	0.4	13.9	44.6			M	5.0	3.1	2.6	4.4
	Two	S	32.7	0.2	8.4	39.1			N	0.9	1.2	1.3	0.7
		M	52.8	0.4	17.7	52.2	Corr				0.61	0.53	0.97
		N	189.0	0.7	40.2	199.2	Iso	One	S	10.2	10.2	7.4	8.1
	Three	S	0.7	0.0	− 1.7	0.8			M	37.6	21.2	21.9	34.4
		M	0.3	0.1	0.6	0.5			N	40.7	37.3	24.3	39.2
		N	9.2	0.2	6.6	9.1		Two	S	7.6	5.6	5.6	7.5
Corr				0.68	0.65	0.91			M	21.8	12.7	13.9	21.0
Amph	One	S	116.0	–	73.5	112.6			N	17.4	14.6	15.2	17.9
		M	177.8	–	169.3	183.2		Three	S	5.7	3.8	4.1	5.6
		N	149.8	–	127.4	153.8			M	48.6	20.3	13.8	43.7
	Two	S	104.9	–	79.6	101.0			N	8.6	8.5	14.9	8.8
		M	187.0	–	149.9	193.4	Corr				0.73	0.60	0.97
		N	141.5	–	115.7	146.9	Poly	One	S	19.5	–	15.0	16.4
	Three	S	76.7	–	62.5	73.6			M	22.9	–	11.3	15.1
		M	210.1	–	125.1	218.9			N	31.4	–	16.4	18.2
		N	126.5	–	88.2	120.9		Two	S	13.6	–	12.1	9.8
Corr					0.81	0.98			M	13.2	–	10.2	10.0
Biv	One	S	161.0	98.2	68.3	202.6			N	28.1	–	14.0	22.3
		M	111.5	43.3	40.2	117.5		Three	S	39.8	–	24.0	30.6
		N	104.9	78.7	81.9	115.7			M	30.9	–	18.8	18.4
	Two	S	68.4	56.3	35.8	82.0			N	40.6	–	25.7	33.1
		M	50.6	34.1	27.2	54.9	Corr					0.47	0.94
		N	93.6	43.9	43.5	110.2	Total	One	S	330.8	–	232.0	346.8
	Three	S	97.9	75.5	47.7	116.8			M	393.5	–	326.9	388.6
		M	72.9	43.0	34.3	83.1			N	383.4	–	375.0	376.2
		N	107.5	70.5	60.8	129.1		Two	S	236.5	–	219.4	242.8
Corr				0.68	0.72	0.96			M	333.3	–	299.6	335.8
Cum	One	S	1.7	2.7	2.5	1.6			N	478.9	–	339.3	499.8
		M	4.9	4.0	3.6	5.2		Three	S	228.7	–	193.9	231.6
		N	3.7	2.2	2.1	3.7			M	370.5	–	258.9	368.9
	Two	S	2.7	2.0	2.1	2.8			N	298.8	–	295.2	302.3
		M	3.2	3.0	2.7	3.3	Corr					0.48	0.95

*Logistic regression fitted values (probability of occurrence) are presented under column H

### Kriging and IDW validation

**Fig. 7:**
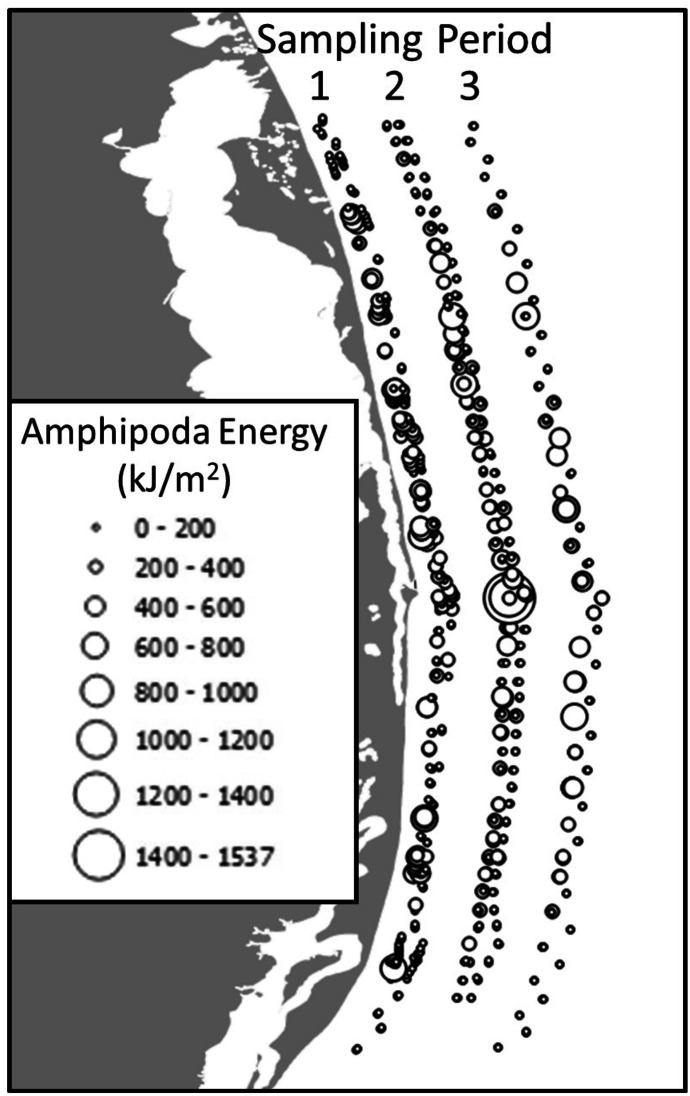
Amphipoda energy density (kJ/m^2^) in the 2015 nearshore study area, Sakhalin Island, Russia. Bubble plots of energy density by sampling period represent replicate values at individual sampling points. The shoreline is added for geographic context and is not an accurate representation of distance from shore

**Fig. 8:**
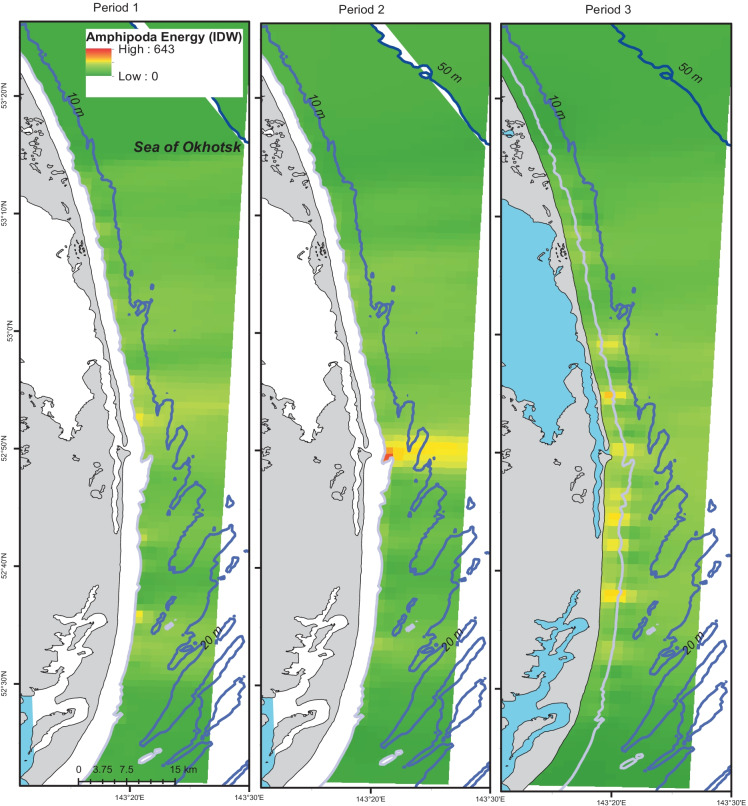
Inverse distance weighting predictions of Amphipoda energy density for the nearshore feeding area Sakhalin Island Russia, 2015

Kriging and IDW interpolations were compared for Amphipoda energy to understand their comparative strengths. Observed data patterns indicated higher Amphipoda energy densities in the middle of the study area, close to shore and in Period 1 with energy density declining over time though very high values were apparent in the middle of the study area in Period 2 (Figs. 2 and 7). High observed Amphipoda energy density also occurred in the south (Fig. 7). Kriging interpolations were higher nearshore in the south and in Period 1 with the peak of energy was just south of the mouth of Piltun Bay (Fig. 2). Kriged Amphipoda energy predictions declined with greater distance to shore. IDW energy interpolations demonstrated higher Amphipoda energy density north of the mouth of the lagoon in Period 1, a peak adjacent to the mouth in Period 2, and an area of elevated energy density throughout the middle of the study area in Period 3 (Fig. 8). The IDW prediction maps encompass a larger area reflecting a representative spatial domain often used for descriptive maps, rather than the restricted domain of the kriging predictions that was limited by spatial–temporal mismatches in predictor value combinations. As a result, IDW predictions extend further offshore and Amphipoda energy interpolations were elevated offshore in Period 2 indicating overprediction of energy relative to distance to shore. Otherwise, IDW and kriging predictions were generally agreeable with higher Amphipoda energy density close to shore and lower values with greater distance to shore. The regression kriging interpolations had larger smoothed areas of high energy density whereas the IDW interpolations more closely reflected localized changes.

## Discussion

### Energy density distributions

Energy content of northern amphipods is low in spring but increases very quickly with the onset of the spring phytoplankton bloom, with smaller changes over mid- to late summer (late July–August) and fall (September–October). Biomass of *Monoporeia affinis*, a dominant in the nearshore Sakhalin Island area, increased sharply ~ 4 weeks after the spring bloom in the northern Baltic Sea and peaked in late July/August with higher biomass into September (Lehtonen, 1996; Lehtonen & Andersin, 1998), a pattern also observed for energy density for *Ampelisca* in the northern Bering Sea (Highsmith & Coyle, 1992). Although early spring changes in energy content occurred prior to the first sampling period of the present study, seasonal patterns of change and presumed peaks in energy content of amphipods and other benthic animals in the nearshore area coincided with seasonal increases in predation (Blanchard et al., 2022; Maresh et al., 2022). Significant variation in energy distributions among the three sampling periods was evident in every regression model in the present study, even though sampling missed the early spring period. In addition to seasonal energy accumulation and growth, temporal differences among sampling periods reflected distributional changes (e.g., migration of sand lance into the study area in Period 2; Blanchard et al., 2022). Except for Polychaeta, Period 3 had the lowest energy densities for all groups with Period 2 highest for Actinopterygii and Amphipoda and Period 1 highest or equal to Period 2 for Cumacea, Isopoda, and total biomass. The declines in energy density across seasons may reflect the influences of respiration and growth combined with summer predation by invertebrates, fishes, and marine mammals, particularly for Amphipoda (a group targeted by gray whales). The one group less likely to be preyed upon, Bivalvia, showed no significant differences among periods while depth effects were very strong, indicating that spatial drivers of variation were dominant for this group. Benthic-feeding predators including fishes and marine mammals regularly crop bivalve necks and consume smaller bivalves whole (Harris et al., 2009; Jewett & Feder, 1980; Paul et al., 1979) but there appears to be fewer predators (e.g., bearded seals and walrus) that target larger bivalves (the older animals usually comprising a large proportion of benthic biomass) in the study area. Polychaeta energy density was the highest in Period 3, but without species information it is not possible to draw inferences because of the numerous life histories within that class. Benthic larvae were found in the water column in June adjacent to Sakhalin Island demonstrating that the timing of early benthic community life history stages is tuned to spring water primary production (Tok et al., 2017).

The scale of spatial structuring in benthic communities and their oceanographic and environmental controls are key influences on predictive power and model fits. Where benthic community patterns, environmental gradients, and spatial correlations are large and smooth over moderate to large spatial scales (oceanographically smooth habitats), model-based kriging can be a powerful and informative tool to model community parameters and uncover patterns (Blanchard, 2015; Blanchard et al., 2013; Grebmeier et al., 2015; Schonberg et al., 2014). It is common, however, for mismatches between spatial scales of change, heterogeneity, sampling, spatial correlations, and predictors to result in significant analytical gaps (Blanchard & Feder, 2014). Gaps are particularly prevalent in oceanographic studies where field seasons are limited and balancing costs with multidiscipline sampling needs can limit data collections. Additionally, exposed habitats such as the nearshore area adjacent to Sakhalin Island can be particularly difficult to model due to high community-level and environmental variability at all spatial scales, as also shown for the coastal North Sea (Armonies, 2000). Here, the GAMMs accommodated the high environmental complexity adjacent to Sakhalin Island associated with distance to shore, water depth, and northing. The complexity was particularly important for Actinopterygii, Amphipoda, Polychaeta, and total biomass where northing by distance interactions were noted. The interactions in the unconditional regressions reflected the changes in bathymetric and seafloor complexity moving alongshore as distance from shore was highly correlated to water depth and other DEM indices of complexity along the narrow shelf. The significant interaction effect for Actinopterygii reflects seasonal migration (higher energy density in the north where they aggregate and particularly in period 2) as well as responses to interannual differences in oceanographic conditions (Fadeev, 2011). For Amphipoda, the interaction reflects the influence of discharges from and oceanographic conditions around Piltun Bay on production with higher energy densities adjacent to the mouth of the bay. Conditional regressions were not as effective in capturing the interactions, as the simple effects of northing and distance were significant for two Cumacea and Isopoda and depth and northing for Bivalvia. The only interaction effect for a conditional regression was the period by northing interaction for Isopoda. The simple effect of depth in the conditional and hurdle models for Bivalvia reflects the strength of the water depth gradient (and associated variations in carbon delivery and sediment structure) on bivalve communities.

The two-part hurdle model was useful for managing zero-inflation of Cumacea and Isopoda where zero values define habitat associations. Substantial improvements in fitted values for hurdle models relative to unconditional GAMMs (models that do not account for the large proportion of zero biomass values) for Cumacea and Isopoda suggested that positive biomass values defined habitats and zeros delimited unsuitable environments (Barry & Welsh, 2002). In contrast, the similarity of the fitted values from the hurdle and unconditional regressions for Bivalvia suggests that zero values occurred within bivalve habitat and no habitat combination was unsuitable. As a result, there was a slight improvement of the unconditional regression over the hurdle model, despite stronger assumption violations (variance heterogeneity due to zero values) with the unconditional approach. Isopods and cumaceans tend to occur less frequently and in patches while bivalves are numerous, the group diverse, and species occur throughout the varying nearshore habitat. Thus, the zero-inflated models appear useful where zero values define habitat characteristics related to patchy biomass distributions, but not where zeros represent within-habitat variability.

The patterns in energy density predictions were similar to biomass patterns noted by Blanchard et al. (2022). Amphipod energy density was higher in the shallow waters (< 13 m) closer to shore while Bivalvia energy density was higher in deeper waters (~ 13–15 m) and at a greater distance to shore. Actinopterygii energy density increased with the seasonal migration of sand lance into the northern portion of the nearshore feeding area during the middle of summer. The latter patterns also represented the strongest trends in the raw biomass analyses of the 2015 data and in prior studies as well (Blanchard et al. 2022; Fadeev, 2011). In contrast to total prey biomass that varied by season and sampling zone (Blanchard et al., 2022), total energy density declined over time across all the study area. The difference arises from correcting for bivalve shell weights in energy density estimates compared to raw wet weight biomass estimates that included shells. Although bivalves are not likely to be a preferred target of grey whales, they are part of the benthic habitats utilized by gray whales and provide a substantial contribution to the energetic landscape of the Sakhalin Island nearshore study area. Finally, the faunal difference in energy associated with the geographic predictors were large, despite the small depth range of ~ 10 m. Demchenko and Fadeev (2011) also demonstrated strong community differences associated with water depth, sediment grain-size, and salinity adjacent to Sakhalin Island (Blanchard et al., 2019; Sobolevskii et al., 2000).

### Modeling

The complexity driving ecological interactions can vary from one area to another within a habitat and can be difficult to model because habitat complexity contributes to ecosystem heterogeneity (de Souza et al., 2013). Spatial–temporal interactions of biological, hydrographic, and oceanographic characteristics with seafloor and shoreline topography can be significant ecosystem influences affecting benthic community characteristics, long-term maintenance of benthic energy hotspots, and thus marine animal distributions (Blanchard et al., 2013; Buhl-Mortensen et al., 2012; Grebmeier et al., 2015). Such variations were expected in the complex nearshore environment adjacent to Sakhalin Island. Potential influences associated with Piltun Bay (e.g., discharge of nutrients from the bay), for example, coincide with increased complexity of the shelf and change in slope. The environmental and physical complexity was reflected in distance to shore, water depth, and the geographic variable northing. As opposed to linear models, GAMMs can better accommodate adjustments for complexity in predictor variables (Guisan et al., 2002; Murase et al., 2009) and particularly for complex interactions that can be modeled through tensor products. Comparisons of GAMMs to multiple linear regression models with two- and three-way interactions suggested a greater simplification, avoidance of collinearity with interactions, and better fits associated with GAMMs using smoothed terms (A. Blanchard unpublished model validation results).

The GAMMS and kriging interpolations in the present study were hindered by the lack of sampling across the full environmental gradient in each sampling period at prediction grid boundaries, a common problem associated with logistical considerations for field studies. IDW maps are not as limited by design mismatches but can produce erroneous and unconstrained estimates outside of observed data or where data points are far apart. While regression approaches provide information concerning the influences of predictors on a response and the ability to make predictions for new covariate values, model-based analyses may not always capture specific, localized changes due to over-smoothing. In contrast, IDW maps can present data patterns for any collection of data points and capture local variations but may not perform well under other circumstances and are descriptive in nature. Here, inverse distance weighted (IDW)–fitted values were faithful for the patterns of the observed data and, in most cases, were more strongly correlated with observed data than other model fitted values. Nevertheless, IDW predictions overestimated energy density for Amphipod in period 2 offshore whereas kriging interpolations were constrained by the water depth gradient. The latter did not, however, express the peak biomass in period 2 as well as the IDW interpolations. The very high interpolated energy content for Bivalvia along the deeper margin of the feeding area represents an overestimation of energy density where energy should decline with greater water depth. Regression predictions are less reliable at boundaries, data extremes, and curve endpoints, all of which can exacerbate edge effects in spatial interpolations. More data points were needed in the present study to correctly shape the relationship at the edge for Bivalvia due to the strong depth/energy density covariance, as noted by Sobolevskii et al. (2000). Poor fits along boundary margins were not noted for other groups. For both methods and as a general rule, the distances among neighboring sampling points need to be small enough and the sampling frame broad enough to correctly model patterns and prevent bullseyes due to too few neighbors or extrapolations with unreliable estimates.

### Prey energy density

Amphipod biomass declines in the Sakhalin Island nearshore feeding area have been large, representing > 30% reductions in energy availability from 2001 to 2015 (Blanchard et al., 2019). Declines are of concern as decreased prey energy density in the nearshore feeding area could adversely affect reproductive female and calf survival, especially when other stressors, like the acoustic stress in 2015, are present. A minimum average Amphipoda wet weight biomass defining a gray whale feeding area appears to range from 60 to 85 g/m^2^ or, using the caloric conversion factor of 5.2, ~ 312–442 kJ/m^2^ (Blanchard et al., 2013, 2019; Brower et al., 2017). Approximately 13% of the nearshore habitat adjacent to Sakhalin Island in 2015 supported amphipod energy densities above 312 kJ/m^2^ and ~ 5% supported amphipod energy densities above 442 kJ/m^2^ in 2015, with about 49% of the habitat having ≥ 312 kJ/m^2^ total energy and 29% ≥ 422 kJ/m^2^. While the nearshore feeding area in 2015 had significantly lower prey biomass than in prior years, juveniles (≤ 8 years of age including calves) appear to be the only demographic group of whales persisting in the nearshore feeding area (Sychenko, 2011). Adults can feed offshore where prey resources are higher and declines have not been as great as in the nearshore area. Average Amphipoda energy density in the offshore feeding area was ~ 688 kJ/m^2^ with a maximum value of ~ 4,706 kJ/m^2^ in 2015 (Blanchard et al., 2019; Demchenko et al., 2016).

### Data gaps

While most gray whales appear to regain adequate body condition during the summer feeding period, there is some concern for unidentified environmental factors that may influence interannual differences in condition (Bradford et al., 2012). Interannual prey biomass declines and spatial variations are particular factors of concern (IUCN, 2019). Stress on arctic marine animals and habitats is highly seasonal and encompasses physical, environmental, biological, and chemical stressors that can be local or widely dispersed (Bard, 1999; Boesch & Rosenberg, 1981; Gray, 1989; Hoekstra et al., 2003; Naidu et al., 2012; Pearson, 1981). In the context of cumulative effects, benthic prey biomass and energy variations in the Sakhalin Island marine environment reflect the aggregate influences of local to broad-scale biological, climatic, environmental, and oceanographic disturbances and stresses (Blanchard et al., 2019). Data are not, however, available to determine the significance of each factor nor their potential effects in the ecosystem. Climate-related effects, for example, influence gray whales through the control of the length of feeding seasons by ice cover that may limit feeding and energy gains (Gailey et al., 2020; Salvadeo et al., 2015) while also driving changes in benthic biomass. Separation of individual drivers, determination of their effects on the benthos, and clarification of strengths of influences on higher trophic levels would be necessary to predict long-term ecosystem changes and could be enabled by targeted oceanographic research programs. The effects of multiple stressors expressed through benthic community variations can also contribute to the aggregate impacts of the stressors for all benthic predators, from commercially important fishes to gray whales. Additionally, how the spatial characteristics of benthic communities in the nearshore, such as patch extents and persistence, relate to gray whale distributions and use by different whale age groups is unknown. Limited information is available to define prey energy density characteristics spatially or temporally, particularly for water depths of < 8 m, which remains a significant data gap.

## Conclusions

High ecosystem complexity is advantageous for benthic communities but difficult to model. Biomass hotspots are driven by interactions of oceanographic characteristics, complex topographies, and life history characteristics of benthic fauna. Energy densities of dominant macrobenthos in northeastern Sakhalin Island varied by sampling period, the latitudinal gradient (northing), and distance offshore/water depth. The interactions in unconditional regression models provide the clearest view of the spatial interactions, with Amphipoda communities peaking around the mouth of the Piltun Bay and Actinopterygii peaking in the northern portion of the study area in period 2 likely due to seasonal migration as well as interannual oceanographic variations. Total energy density reflected the changing energy distributions of Amphipoda and Actinopterygii. A key benefit of the spatial-regression approach applied here was the incorporation of GAMMs to better model complex relationships providing the means to incorporate smoothed-functions for predictors while accommodating zero-inflation in model-based kriging. This GAMM-based approach was useful for accommodating the high environmental and biological complexity often encountered in marine biological communities. As a complementary statistical analysis, spatial regression modeling provides a fuller picture of biomass changes, compared to community-level analyses alone.
